# Natural capital approaches for the optimal design of policies for nature recovery

**DOI:** 10.1098/rstb.2022.0327

**Published:** 2024-06-10

**Authors:** Brett Day, Mattia Mancini, Ian J. Bateman, Amy Binner, Frankie Cho, Anthony de Gol, Henry Ferguson-Gow, Carlo Fezzi, Christopher Lee, Lorena Liuzzo, Andrew Lovett, Nathan Owen, Richard G. Pearson, Greg Smith

**Affiliations:** ^1^ Land, Environment, Economics and Policy Institute, Department of Economics, University of Exeter Business School, Exeter EX4 4PU, UK; ^2^ Centre for Biodiversity and Conservation Science, School of Earth and Environmental Science, University of Queensland, Brisbane, QLD 4072, Australia; ^3^ School of Environmental Sciences, University of East Anglia, Norwich NR4 7TJ, UK; ^4^ Centre for Biodiversity and Environment Research, University College London, Gower Street, London WC1E 6BT, UK; ^5^ Department of Economics and Management, University of Trento, via Inama 5, I-38122 Trento, Italy; ^6^ CSIRO, Castray Esplanade, Battery Point, Hobart, Tas 7004, Australia

**Keywords:** natural capital modelling, ecosystem services, economics, valuation, nature recovery

## Abstract

By embedding a spatially explicit ecosystem services modelling tool within a policy simulator we examine the insights that natural capital analysis can bring to the design of policies for nature recovery. Our study is illustrated through a case example of policies incentivising the establishment of new natural habitat in England. We find that a policy mirroring the current practice of offering payments per hectare of habitat creation fails to break even, delivering less value in improved flows of ecosystem services than public money spent and only 26% of that which is theoretically achievable. Using optimization methods, we discover that progressively more efficient outcomes are delivered by policies that optimally price activities (34%), quantities of environmental change (55%) and ecosystem service value flows (81%). Further, we show that additionally attaining targets for unmonetized ecosystem services (in our case, biodiversity) demands trade-offs in delivery of monetized services. For some policy instruments it is not even possible to achieve the targets. Finally, we establish that extending policy instruments to offer payments for unmonetized services delivers target-achieving and value-maximizing policy designs. Our findings reveal that policy design is of first-order importance in determining the efficiency and efficacy of programmes pursuing nature recovery.

This article is part of the theme issue ‘Bringing nature into decision-making’.

## 1. Background

Faced with the twin crises of biodiversity loss and climate change, the critical importance of nature to human society is being increasingly recognized by the global community. The potential costs of inaction are staggering. From 1997 to 2011 the OECD estimates that the world lost US$4–20 trillion per year in ecosystem services on account of land cover change [1]. Climate change, it is estimated, will shrink global GDP by 5% by 2050, rising to 13% by 2100 [2]. Like many other nations, policy-makers in the UK have begun to formulate plans to address these challenges. The UK has made legally binding commitments to achieving net zero greenhouse gas emissions by 2050 [3] and to address biodiversity loss by 2030 [4]. In both cases, nature recovery is seen as a key part of the solution. Indeed, to meet these goals the UK government has made commitments to invest £750 million in tree-planting and peatland restoration, to protect 30% of the UK's land and sea for nature and to transform agricultural support schemes to incentivize farmers to deliver environmental improvements [5,6]. While there is undoubtedly ambition to support nature recovery, this paper addresses the question of how economic methods, particularly developments in the application of the natural capital approach, can aid decision-makers in delivery. How should we design feasible policies to make the best use of the limited public funds available to realize nature recovery?

The natural capital approach is a way of thinking about the natural environment in economic terms [7,8]. In essence, nature is regarded as a source of myriad ecosystem services (for example, carbon sequestration, flood mitigation and pollination) that deliver benefits to humans in society and, in that regard, are no different from the services provided by private companies and public agencies. Moreover, the natural capital approach advocates the use of non-market valuation to allow the benefits delivered by ecosystem services to be quantified in monetary terms. For policy-makers, the valuation of ecosystem services is of critical importance in decision-making. Take, for example, the object of interest of this paper: interventions that establish new habitat for nature recovery. Valuation allows the potentially numerous environmental changes that arise from that intervention to be aggregated to a single metric of social value. Moreover, that value can be weighed against the other costs and benefits of a habitat creation project to assess whether society enjoys a net gain from its adoption. Likewise, valuation allows contrasting interventions offering different portfolios of environmental change to be compared on the same metric. In our case, such information allows policy-makers to choose which types of habitat should be created in which locations to ensure that scarce public funds are used efficiently to deliver the greatest benefit for society.

Use of the natural capital approach is increasingly advocated in government decision-making, including in the UK where natural capital principles underpin guidance on policy and project appraisal [9,10]. While straightforward in principle, application of the natural capital approach in practice is made difficult by a number of factors. First, environmental and economic systems are complex. To properly evaluate the changes in ecosystem service flows that arise from, say, a habitat creation project, requires tracing impacts through complex, context-specific and inter-linked environmental systems to their consequences for humans in equally complex, context-specific and inter-linked economic systems [11]. Indeed, to cope with that complexity natural capital analyses have come to increasingly rely on sophisticated spatially explicit integrated environment–economy models^1^. In this paper, for that purpose we introduce and apply the Natural Environment Valuation (NEV) model suite, a set of integrated environment­–economy models that quantify and value ecosystem services across the UK.

A second key complexity in the application of the natural capital approach concerns the valuation of ecosystem services. For many of those service flows, values can be estimated through application of well-established methods of non-market valuation [17–19]. In this study, we refer to those as *monetized services*, a set of services that include carbon storage, recreation, flood damage mitigation and the quality of water abstracted at treatment works. For other service flows, however, little consensus exists regarding how, or even what particular measure of that service should be valued. We describe these as *unmonetized services*. In the context of our study on nature recovery, the most significant service flows that fall into this category are those arising from biodiversity. While our models allow us to quantify changes in the occurrence of species, the numerous routes through which such changes impact ecosystem functioning and thence the supply of myriad ecosystem services essential for human well-being are so complex that, as yet, no accepted approaches exist to attribute them with economic value [20,21]^2^. As such, a key question for application of the natural capital approach in decision-making is how these unmonetized ecosystem services can be accommodated in the decision process.

A third complexity in the application of the natural capital approach to decision-making is that understanding the social value of environmental changes is only part of the information set required by decision-makers to form policy. Again, the problem of habitat creation for nature recovery serves to illustrate the point. Through valuing the ecosystem service flows delivered by different habitat-creation projects, natural capital approaches might allow a decision-maker to answer the important question of which habitats to establish in which locations to deliver the most benefits to society. Indeed, the natural capital approach has been used extensively to answer questions of this ilk; for example, in identifying optimal locations for conservation areas [23,24], agri-environment interventions [25], greening of urban environments [26], afforestation [27], renewable energy infrastructure [28], freshwater allocation [29] and flood risk interventions [30]. Of course, in the real world where land is owned by private agents, policy-makers are rarely in a position to dictate exactly how land is used. Indeed, in practice, policy-makers are constrained to a limited set of feasible and politically acceptable policy instruments that, for example, might offer private land owners incentive payments to establish new natural habitats on their land. The key information that decision-makers require, therefore, may not be how land is best used for nature recovery, but how is policy best designed to deliver nature recovery.

In this paper, we consider each of these complexities in the context of designing policies to deliver nature recovery in England. The potential policy space consists of various forms of payment offered to landowners to incentivize reversion of farmland to different natural habitats. We embed our ecosystem service valuation model (NEV) inside a policy simulator that predicts how landowners across England will respond to a particular payment format. This combined model allows us to simultaneously assess scheme uptake, scheme cost and the aggregate value delivered in monetized ecosystem services, as well as to quantify changes in unmonetized ecosystem services. In this regard, our work is similar to others who have explored pricing strategies and their impact on scheme uptake using the natural capital approach [31–33]. Moreover, we further embed that policy simulator in an optimization framework. As such, our set-up allows us to identify policy formats and payment schedules that deliver the greatest net value to society given a budget-constrained scheme.

Of course, optimizing net value as delivered by monetized ecosystem service flows ignores impacts on unmonetized service flows. In our work, we imagine that policy-makers choose to express societal preferences regarding unmonetized service flows by setting quantity targets for their delivery. That strategy mirrors policy practice in the UK, which has committed to legally binding biodiversity targets [4]. With this addition, we can again use our modelling framework to identify policies that maximize delivery of benefits from monetized ecosystem services, subject to the constraint that the policy also delivers the target level of improvements across biodiversity indicators. Similar to [34,35], these analyses allow us to quantify trade-offs across ecosystem service provision when those services cannot be measured in commensurate units: in our case, this would comprise answering the question of what value we must give up in monetized ecosystem services to achieve the policy-maker's desired level of gains in biodiversity.

Importantly, pricing instruments that are effective at delivering to one measure may not necessarily be effective at delivering to another. Indeed, in our case we find that the more precisely focused our pricing mechanism is on delivering on monetized services, the less effective it is at delivering on biodiversity gain targets. Accordingly, we go one step further and consider extending policy instruments to include prices that incentivize delivery of unmonetized services.

Developing a modelling framework through which we can explore optimal policy design using the natural capital approach allows us to examine a number of important policy-relevant questions and contribute to a variety of literatures. First, our work contributes to the growing literature on policy simulation and optimization in the natural capital framework [34–36]. Moreover, we are able to explore the efficiency properties of different policy designs, contrasting current UK policy instruments with designs that optimally price the activity of establishing habitat and those that optimally price the environmental or ecosystem service outcomes of that activity. In that regard, our work contributes to the literature examining pricing strategies in schemes incentivizing delivery of natural capital and contrasting activity-based and outcome-based incentive payments [37–41]. Indeed, we provide insights as to the magnitude of the efficiency gains that might be realized from adopting different pricing policies in a national scheme targeting habitat creation for nature recovery. Our third area of contribution pertains to the application of natural capital approaches to designing policies seeking to deliver both monetized and unmonetized ecosystem services. While previous authors have adopted multi-objective optimization techniques to appraise the trade-offs in prioritizing one service flow over another [34,35], our work focuses on policy designs that deliver target levels of unmonetized service flows while optimizing delivery of monetized service flows. Moreover, we show that extending policy instruments to directly reward the delivery of unmonetized ecosystem services allows us to identify target-achieving and value-maximizing policy designs.

## 2. Methods

### (a) Case study

Our examination of policy design using natural capital approaches is pursued in the context of a case study of policies seeking to incentivize landowners to establish natural habitat on farmland in England. Loosely based on UK government agri-environment policy, we examine a commitment to spend £1 billion of public money with the objective of delivering the most value in environmental improvements from that expenditure.^3^

This simulated scheme considers two natural habitat types, woodland and semi-natural grassland that could be established in most agricultural settings across England. The former is taken to be planted in a 60:40 mix of native broadleaf to conifers and managed for timber production, and is reflective of UK government plans to significantly increase forested landcover in the UK in pursuit of its net-zero carbon emission commitments. The second habitat, semi-natural grassland (SNG), is unimproved, species-rich permanent meadow providing a low yield hay crop and potentially grazed at low intensity to control woody plant growth. While these were once abundant, the UK has experienced a 97% loss in such wildflower meadows since the 1930s [43]. Since estimates of the benefit flows from recreational access to the countryside suggest this may be an important source of value [44], our policy also presents landowners with the option of choosing to open up their newly created habitat to the public for recreational access. The scheme we simulate, therefore, offers eight different options, defined by habitat type and recreational access and differentiated across previous agricultural use of land.

Our analyses are performed on a 2 km grid across England. Within each 2 km grid square we use landcover data [45] to identify the extent of farmed land under either permanent pasture for livestock grazing or used for arable cropping. We exclude farmed land used for high-value horticultural agricultural activities. We assume that the arable and pasture land in each cell represent separate choice units over which an independent landowner makes profit-maximizing farming decisions. We describe these grassland and arable areas as parcels and those parcels become the basic unit of our analysis, with the landowner of each parcel responding to the incentives presented to them by a policy instrument and choosing whether to commit that land to one of the possible habitat-creation options. Excluding cells with over 50% urban landcover, our analysis comprises 59 648 such land decision units.

Current and past agri-environment schemes in the UK have adhered to the requirements of the EU's Common Agricultural Policy and adopted an ‘income foregone plus costs' payment model [46]. Under that model, landowners are offered a flat-rate payment, with payment levels for each land management activity in the scheme designed to reflect the ‘typical’ agricultural income foregone and the costs incurred in pursuing that activity. The base case policy examined in our simulated nature recovery scheme replicates this payment methodology. We estimate the agricultural income foregone and the costs of delivery associated with pursuing a habitat-creation option on each parcel and then fix the payment level offered in the scheme for that option at the median of the resulting distribution of costs per hectare.

The UK's withdrawal from the EU has ignited a policy discussion on whether cost-based, activity payments should be replaced by alternative payment models potentially rewarding the delivery of desired environmental outcomes [4,47]. Our analyses contribute to that discussion by simulating a series of policies that span the range of alternative instruments under consideration in that on-going debate. One set of such instruments resemble the current policy in paying landowners for the action of pursuing an option. Rather than basing payments on typical costs, however, we explore the benefits of choosing payment rates so that they best deliver on desired environmental outcomes. By contrast, in payment-by-outcome instruments, landowners are offered flat rate prices per unit of environmental outcome delivered by their project. With these schemes, the payment received by a landowner is the sum of the payments they are due across the array of environmental outcomes that change on account of their chosen habitat-creation project.

### (b) Natural capital modelling

Our research is enabled by a set of spatially explicit, environment­–economy models collectively termed the Natural Environment Valuation (NEV) modelling suite. Each NEV model quantifies and values changes in ecosystem services arising from land use change (LUC) in the UK [44,48,49]. We provide a detailed description of the different NEV model components in Appendix 1 of the Electronic Supplementary Materials. Here we summarize key elements of the modelling that are central to understanding our subsequent policy simulations.

#### (i) Scheme option costs

The NEV farm model, derived from a spatially explicit analysis of physical environment, climate, economic and policy data from the 1960s to the present day, allows us to predict a time path for agricultural activity on each land parcel assuming that the climate follows a medium stabilization pathway compatible with a 2.8°C global mean temperature rise by the end of the century [50]. We use the same climate time series to drive all ecosystem service models from the NEV suite. Current margins on food production are used to approximate returns to agricultural activity on each parcel over a 100 year time horizon from 2020. Then, following a procedure mirrored in similar calculations for all NEV ecosystem service models, we convert the 100 year time series into an equivalent annuity and finally calculate the net present value (NPV) of foregone returns to agriculture from permanent land use change assuming a 3.5% discount rate. Indeed, all our analyses are in terms of NPVs calculated to a 2020 base year and expressed in terms of 2020 prices.

For a landowner to consider pursuing a habitat-creation project on their agricultural land parcel we assume that the incentive payment they receive must exceed this estimate of foregone income from agriculture, plus the net costs of establishing and maintaining the habitat as well as a mark-up of 15%. That 15% mark-up on costs is included to reflect additional private transaction costs from scheme participation [51,52]. The costs of establishing woodland are associated with planting and management activities and are taken from the UK Forestry Commissions FIAP model [53]. Projects that additionally allow public access also incur costs through the creation of a path network and provision of car parking to accommodate peak hourly recreational visitation by car to the site. The latter is estimated from the NEV recreation model, which also predicts the value of annual visits [54,55].

#### (ii) Scheme option benefits

If pursued, each possible habitat-creation project would precipitate changes in environmental systems. Using NEV's environmental system models we are able to quantify the consequences of those changes on an array of environmental outcomes; particularly in yields from terrestrial ecosystems, storage and emissions of greenhouse gases, changes in water quality and peak flows in surface water and in the composition of the biotic community. The extensive array of environmental outcomes captured in our analyses are listed in the second column of table 1 and described in detail in Appendix S1 of the electronic supplementary materials. Notably, we provide a comprehensive accounting of greenhouse gases, capturing changes in carbon stored in biomass and in soils. Likewise, we quantify both the domestic emissions avoided from the farming activities displaced by the habitat-creation project, and also use current trade patterns to estimate the increase in international emissions resulting from food production displaced overseas on account of loss in UK agricultural output. With regards to biodiversity, we employ a set of presence/absence models that predict the occurrence of 428 pollinator species and 386 other species featuring in the UK Joint Nature Conservation Committee (UKJNCC) priority species indicator. The models operate at a 2 km grid resolution and can be used to predict changes in species presence on account of the change in composition of land use within a cell arising from a habitat-creation project. WFD, Water Framework Directive.

**Table 1.** Ecosystem service flows quantified and valued by the Natural Environment Valuation (NEV) model suite.

environmental system	environmental outcome	ecosystem service value
terrestrial	food yield from farmland (tonnes of each product)	net returns from food production
	wood product yield from woodland (m^3^ timber)	net returns from timber production
	hay yield from semi-natural grassland (tonnes dry matter)	net returns from hay production
	woodland recreational site (hectares)	value of outdoor recreation activity
	semi-natural grassland recreational site (hectares)	value of outdoor recreation activity
atmospheric	emissions from farming (tonnes CO_2_e)	value of carbon sequestration
	emissions from displaced food production (tonnes CO_2_e)	cost of carbon emissions
	carbon stored in soils (tonnes CO_2_e)	value of carbon sequestration
	carbon stored in trees and wood products (tonnes CO_2_e)	value of carbon sequestration
hydrological	nutrient concentrations (micrograms/litre)	nutrient treatment costs at water supply plants
	reduction in peak flow (litres/day)	mitigation of risks of property damage from flooding
	ecological status (WFD classification)	recreational value of improvements in river ecological status
	ecological status (WFD classification)	non-use value from improvements in river ecological status
biotic community	pollinator species occurrence (species richness index)	value of yield from insect-pollinated crops
	pollinator species occurrence (species richness index)	aesthetic value of insect-pollinated wild flowers
	pollinator and priority species occurrence (species group prevalence)	—

For the majority of environmental outcomes that we are able to quantify with the NEV model suite, we are also able to apply methods of non-market valuation to estimate the value of the change in associated ecosystem service flows. The ecosystem service values used in our analyses are listed in the third column of table 1 and detailed in electronic supplementary materials, appendix S1. We capture both values enjoyed on the production side of the economy and on the consumption side. For example, the NEV hydrological models allow us to estimate the savings in drinking water processing costs arising from reductions in nutrient concentrations in surface water abstracted at treatment plants downstream of a habitat creation project. Likewise, we estimate the value gains enjoyed by consumers in recreation and non-use from improvements in the ecological condition of rivers arising from those same reductions in nutrient concentrations. For biodiversity we estimate both the value to farming of increased pollination services in high-value horticulture and also the value to consumers of increased prevalence of insect-pollinated wild-flowers.

While our models are able to quantify changes in the occurrence of species the myriad routes through which those changes deliver ecosystem services to society are so complex that we do not have a good way of attributing them with economic value. In the absence of value estimates, we therefore simulate policies that seek to achieve target levels of improvements in biodiversity. To form those targets we organize our 814 species into eight groups (hoverflies, bees, lower plants, lichen, gastropods, arthropods, fish, shellfish) designed to provide broad coverage of British taxonomic groups. We calculate the quantity of cells in which each species was predicted to be present across England in 2020 and then sum those to give a baseline ‘prevalence score’ for each of the 8 groups. The policy target would then be to invest in habitat creation projects that act to increase prevalence by at least some percentage across all species groups by 2030.

For many of the models in the NEV model suite, the benefits of land use change in one parcel impact on the benefits realised from land use change in another. A case in point is the recreation model. Establishing a new natural area with recreational access in one parcel not only increases recreational benefit flows from that parcel, but also acts as a substitute for recreational areas in neighbouring parcels reducing their benefit flows. Such inter-parcel dependence in benefits greatly increases the complexity of the combinatorial optimization problems that we need to be able to solve when examining outcomes under different scheme designs. As documented in the electronic supplementary material, therefore, for such models we approximate the benefits of land use change in each parcel using an average marginal benefit measure. While only an approximation to the true benefits, using these approximations simplifies analyses by ensuring that benefit measures are independent across parcels.

### (c) Policy simulations and optimization

We imagine a decision-maker seeking to maximize the aggregate benefits delivered by the ecosystem service changes arising from habitat-creation projects. The policy-maker has a fixed budget to spend and does so by offering landowners payments for pursuing a habitat creation project on their land parcel. The decision-maker's problem is how best to design the structure of payments in their scheme to deliver the most environmental value for the scheme budget.^4^

Applying the principles of the natural capital approach, we use the NEV models to predict the sum of ecosystem service value changes for each project option on each land parcel. Clearly, such aggregate value estimates reflect benefit flows from monetized ecosystem services, but fail to capture the potentially important contributions from biodiversity, which we are unable to value. We consider that omission subsequently.

In theory, the very best that the policy-maker could do would be to pay landowners an amount that exactly covered their costs of project delivery and, paying only that amount, select the set of projects that deliver the most aggregate value achievable within the budget. As we show in appendix S2 of the electronic supplementary materials, that problem can be formulated as a multiple-choice knapsack problem and, in our simulations, we use an algorithm proposed by Pisinger [56] to solve for the set of projects that deliver that in-theory, upper-bound scheme value.

Current UK agri-environment policies generally offer farmers a flat-rate payment per hectare based on the typical costs of option delivery [57]. We therefore calculate the median cost per hectare for each of our permanent LUC options across all land parcels in England. Presented with those flat-rate prices, landowners who can profit from the scheme choose to volunteer their parcel for the option that returns them the most surplus. Offering a price that 50% of farmers would accept for each option results in uptake requiring payments in excess of the scheme budget. As such, we simulate this policy as a first-come, first-served scheme randomly ordering the arrival of landowners' applications and selecting parcels up to the point at which the budget is exhausted. Our estimates of the aggregate value delivered by this scheme come from averaging the aggregate ecosystem service value delivered by 1000 simulated runs of this scheme.

Rather than pegging flat-rate payments to costs, the natural capital approach suggests that it would be more efficient to identify flat rate payments per hectare of each option that maximize the aggregate value delivered by the scheme. To examine the efficiency gains from optimal flat rates for activities, we turn to methods of Mixed Integer Programming (MIP). As described in the Appendix 2, this problem is a variant of the Unit-Demand, Envy-Free pricing problem [58] which we apply to our data and solve using the CPLEX software [59].

Greater efficiencies still may be attainable by switching the focus of payments from the activity of creating habitat to paying directly for the desirable outcomes that arise from that planting activity. Focusing payment on environmental outcomes rather than on activities ensures that the scheme only encourages projects where they deliver environmental improvements.^5^ Drawing on the list of environmental outcomes from table 1 we simulate a scheme that offers flat rate prices for each unit of improvement across eight different environmental outcomes including tonnes of CO_2_e sequestered, phosphate and nitrate concentrations in surface water, reductions in peak flows, pollinator species richness, areas of different new habitat accessible for recreation and areas not accessible. Again, we solve for the set of environmental outcome prices that deliver the greatest aggregate value for the budget using MIP.

An alternative form of outcome-based payment is one where landowners are rewarded for the value of the ecosystem services they deliver. Again, *a priori*, such a policy design has the potential to deliver efficiency gains since it directs payments to projects where the environmental change resulting from habitat creation generates the most value. The prices we use in our simulation are those identified in the final column of table 3 and include a price per unit value of recreation activity, carbon sequestered, water treatment cost avoided, flood damage cost avoided, recreation and non-use from improved river ecological status, yield of insect-pollinated crops and from the prevalence of insect-pollinated wild flowers. We again solve for the set of prices that deliver projects offering the greatest aggregate value within the budget.

**Table 3.** Prices offered to landowners in the payment by activity scheme simulations.

prices	payment mechanism			
	cost-based prices	optimal prices		
		budget constrained	+biodiversity constrained	+biodiversity pricing
*activity (£ per hectare)*				
arable to SNG, access	12 266	8973	8187	7797
pasture to SNG, access	11 422	2914	5475	5255
arable to woods, access	23 312	20 406	19 340	19 287
pasture to woods, access	22 279	0	0	14 477
arable to SNG, no access	11 834	0	0	7516
pasture to SNG, no access	11 096	2747	5310	5088
arable to woods, no access	22 951	19 607	0	0
pasture to woods, no access	21 945	0	0	14 269
*biodiversity (£ per additional species presence in a 2 km cell delivered by project)*				
bees	—	—	—	8
hoverflies	—	—	—	51
arthropods	—	—	—	0
fish	—	—	—	65
gastropods	—	—	—	1099
lichen	—	—	—	0
lower plants	—	—	—	190
shellfish	—	—	—	383

While adoption of the Natural Capital approach allows us to consider the efficiency gains that arise from carefully designing policy measures, the simulations discussed so far ignore the benefits from biodiversity that we are unable to reliably monetize. Accordingly, we imagine the UK government setting a target amounting to a 15% improvement in the prevalence of species in our eight species groups. We re-run each policy simulation searching for a policy design that maximizes aggregate ecosystem service value flows while delivering the desired improvements in biodiversity.

While pricing by environmental outcome and even more so pricing by ecosystem service, allows us to more precisely target projects that deliver enhanced aggregate value from monetized ecosystem services, there is no guarantee that those pricing instruments are effective at delivering projects in locations that best deliver increases in species prevalence. Our final set of simulations explore the possibility of including further prices that directly reward delivery of species prevalence in each subgroup. Formally, this amounts to including prices that not only target measures that enter the policy-maker's objective function (aggregate ecosystem service value) but also the constraints they place on maximizing that function (improvements in species group prevalence).

## 3. Results and discussion

The central results of our policy simulations are provided in table 2 which reports on the value-for-money achieved by the different scheme designs. Value-for-money is calculated from the point of view of the policy-maker as the increase in aggregate ecosystem service value flows arising from the habitat change projects funded by the scheme divided by public money spent. In all cases that spend was more than 99.8% of the budget of £1 billion. The in-theory, upper bound of this value-for-money statistic is 3.329, which can be interpreted as indicating that £3.33 of ecosystem service value is delivered by every £1 spent through the scheme.

**Table 2.** Value for money delivered by the budget-constrained scheme under different payment mechanisms and when seeking to achieve biodiversity targets.

payment mechanism	scheme value for money (value per £ spent)		
	budget constrained	+biodiversity constrained	+biodiversity pricing
in-theory upper bound	3.316	—	—
payment by activity:			
cost-based prices	0.860	infeasible	—
optimal prices	1.105	0.874	0.890
payment by outcome:			
optimal prices for environmental outcomes	1.785	1.785	1.785
optimal prices for ecosystem services	2.628	infeasible	2.494

Our first important finding is that when adopting current UK government cost-based pricing practices, the scheme does not manage to break even. From table 2, we observe that under that pricing mechanism, for every £1 spent, only £0.86 is delivered in ecosystem service value flows, amounting to only 26% of the in-theory upper-bound.^6^

Simply offering landowners flat-rate payments per hectare based on the typical costs of option delivery proves inefficient on account of three factors. First, it ignores the possibility that this choice of prices rewards farmers with payments beyond what is required to satisfy their need for compensation. In our simulation, the average profit (payment over cost) received by farmers selected through this scheme was some £1861 per hectare. Second, pricing based on the typical costs of each option ignores the fact that different options may deliver different levels of ecosystem service enhancement. In the England data, averaging across all possible projects we find that the value of those enhancements per hectare differs across options by an order of magnitude. Inefficiencies arise with pricing based on the typical costs of options because scarce public funds are not differentially directed to those activities that provide the best returns on investment. Third, pricing by activity means that within an option, the projects that will be attracted to the scheme will be those that can supply that option's activities relatively cheaply. If activity cost and ecosystem service enhancement are perfectly negatively correlated then this is not an issue; relatively cheap projects are also valuable projects. However, perfect negative correlation does not characterize the England data. Across the eight options in our analysis, we find that the correlation between per hectare project costs and values ranges from a low of −0.373 to a high of 0.301. Inefficiencies arise, therefore, some low cost projects will be funded despite offering very low value while relatively high cost projects offering very good value will not.

A better understanding of the extent of these inefficiencies can be gathered by using the policy-optimization techniques advanced in this research. Continuing to offer a price per hectare for each of the eight scheme options, we solve for those prices delivering the greatest aggregate ecosystem service value flow within the budget. By choosing option prices optimally, we are targeting the first two inefficiencies described above; those arising from over-rewarding farmers for an activity and those arising from not distinguishing across options by the ecosystem service values delivered by those activities.

From table 2, it is clear to see the advantage of adopting an intelligent pricing rule. The scheme now returns 29% more value than with cost-based pricing and more than breaks even, offering a value for money ratio of 1.105.

The optimal prices for activities are listed in the third column of table 3, where they can be contrasted with the cost-based prices listed in the second column. In all cases, the value-optimizing activity prices are lower, often substantially lower. In general, reducing prices ensures the policy avoids over-rewarding landowners. The average payment over cost received by farmers is now only £654.12 per hectare, a third of that under cost-based activity pricing.

Observe that the optimal prices now strongly differentiate across activities, dropping prices for certain activities to zero and focusing payments on those activities delivering the best value for the investment of public money. In our simulations, the optimal pricing structure clearly favours projects planting woods on arable land (table 3) an activity which invariably delivers substantial greenhouse gas sequestration benefits displacing relatively high emissions agriculture and offering good potential to store sequestered carbon in soils and biomass. Indeed, almost 84% of the value flow realised by this scheme is from greenhouse gas removal (see electronic supplementary materials, appendix S3, table SM7). The reason why that pricing structure optimizes scheme value can be found in the heterogeneity of values delivered by different scheme activity options. While one LUC project may, for example, offer significant flood protection or recreation benefits on account of its location, this pricing mechanism cannot differentiate that project from another offering identical LUC but in a location that delivers none of those service flows. By contrast, the greenhouse gas removal benefits of planting trees on arable land are relatively homogeneous across space. As such, when constrained to pay by activity, our simulations indicate the best pricing strategy is to focus spending on activities that offer uniformly positive returns across space eschewing other activities that may return high value in one location but little in others.

Again, insights into the inefficiencies arising from schemes that pay by activities are provided by simulating schemes adopting the alternative paradigm of paying by outcomes. Referring to table 2, we find that a scheme offering optimally determined prices for an array of environmental outcomes delivers scheme value for money of 1.787, approximately half of the theoretically achievable upper bound. Going one step further and paying directly for the value of each ecosystem service flow delivered by a project enables the scheme to achieve value for money of 2.628, which amounts to 79% of the upper bound and a value flow that is over 3 times that achieved by currently applied cost-based pricing.

The efficiency gains of payment by outcome policy designs are achieved by presenting a payment schedule that most rewards high-value projects. The flexibility that outcome payments introduce allows the mechanism to target projects that provide value through any of the ecosystem service channels. In contrast to the payment by activity designs where the vast majority of value arose from greenhouse gas removal services, under the optimally priced payment by ecosystem service design, significant value flows are also realised from projects delivering recreational service flows (47% of entire value delivered), pollination services to agriculture (13%), flood mitigation services (9%) and wild flower abundance (6%) (see electronic supplementary materials, appendix S3, table SM7).

An interesting feature of the payment for ecosystem service prices determined through our optimization algorithms is that for some value flows the prices paid exceed 1 (see electronic supplementary materials, appendix S3, table SM9). Upon first examination, such pricing appears irrational. Why pay more than £1 for each £1 of value delivered through a particular ecosystem service? In point of fact, projects deliver non-separable bundles of services which exhibit complex patterns of correlations across both different services and project costs. Through those correlations paying highly for one service may encourage cheaper delivery of some alternative and highly valuable service flows.

When we extend the policy scope to include the achievement of biodiversity targets, our policy simulations reveal further interesting patterns (column 3 of table 2). We find that delivering the target 15% gain in each species group is simply not achievable with the cost-based activity payments currently used in UK agri-environment schemes.^7^ Moreover, at the activity prices that optimize delivery of monetized ecosystem services, 7 of the 8 biodiversity gain targets are not met (see electronic supplementary materials, appendix S3, table SM10). Using our optimization algorithms, however, we are able to identify activity prices that achieve the targets (column 4 of table 3) though to do so requires a significant sacrifice in delivery of monetized ecosystem services: value for money is 0.874 compared to 1.105 without the biodiversity constraint.

The same is not true with the payment for environmental outcomes policy. Here the prices that optimize the delivery of aggregate value of monetized ecosystem services also achieve biodiversity gains that meet the targets across all species groups. That stands in stark contrast to the payment for ecosystem services policy. Here we find that both the target gains for lichen and those for lower plants are not achieved at the value-optimizing prices (see electronic supplementary materials, appendix S3, table SM10). Indeed, our optimization algorithms reveal that there is no combination of prices for ecosystem services that is able to incentivize projects to join the scheme that achieves all eight biodiversity targets. The key insight provided by this observation is that focusing our pricing mechanism more intently on the delivery of monetized ecosystem service flows, in no way guarantees that we will also be able to deliver sufficient non-monetized ecosystem service flows. Our simulations indicate that the degree of correlation between monetized ecosystem services and unmonetized species-group prevalence is insufficient to use the former to target delivery of the latter.

Our final set of investigations explore how extending policy mechanisms to admit pricing of the measures that make up the biodiversity targets allows for more efficient delivery of those targets. For the payment by activity scheme, optimally choosing that extended array of prices (see column 5 of table 3) results in only minor gains; the value for money of the scheme with regards to monetized ecosystem services increases from 0.874 to 0.890. In a similar vein, since the biodiversity targets are achieved when choosing optimal prices for environmental outcomes to maximize aggregate ecosystem service value, adding prices for biodiversity outcomes does nothing to improve the efficiency of the mechanism in reaching those targets. In the case of pricing for ecosystem services, however, pricing biodiversity outcomes is essential to allowing the mechanism to achieve the biodiversity targets. As shown in electronic supplementary materials, table SM9 (appendix S3), the optimal price array includes fairly substantial payments for biodiversity outcomes allowing the mechanism to achieve the target and deliver a value for money with respect to monetized ecosystem services of 2.494. Again, we observe that achieving biodiversity targets comes at a cost; the value of monetized ecosystem services delivered by the scheme falls by some 5%.

## 4. Concluding remarks

While the need for action on nature recovery is now widely accepted (witness the UK's 25 year environmental plan [6], the EU's biodiversity strategy for 2030 [60] and the Biden administration's ‘America the Beautiful’ initiative [61]), how best a programme of action to deliver that goal should be implemented remains an open question. This paper examines the contribution that advances in the natural capital approach might make to the task of designing the required policy mechanisms. In particular, we focus on extensions to standard natural capital analyses that seek to simulate landowner participation in schemes incentivizing habitat creation and show how optimization methods can be used to identify policy mechanisms that efficiently deliver to policy-maker goals. Our research reveals a number of important quantitative and qualitative insights.

Our first key finding is that poorly designed policies for nature recovery may result in net losses in value to society. Simulating, a policy mirroring the currently accepted methodology for pricing incentives for habitat creation projects in the UK, we find that the policy delivers relatively low-performing projects. Not only do these projects fail to deliver monetized ecosystem service improvements of greater value than the public money spent on them but they also fail to achieve targets for delivery of unmonetized biodiversity improvements. Moreover, by embedding natural capital models within a policy simulator we are able to show how a simple change to that policy that efficiently adjusts pricing points for this instrument results in a 29% uplift in value and ensures that society receives a net gain in value from its investment.

Our modelling environment allows us to go further and explore alternative pricing instruments. In our study we focus on alternatives that pay landowners according to the desired outcomes their projects deliver.^8^ We show that the magnitude of the possible gains of moving from payments for activity instruments to payments for outcome instruments are very significant. The value realised by the latter is 2.4 times greater than the former, and some 79% of the theoretically achievable upper bound.

Our work also sheds light on the magnitude of the trade-offs that result from seeking to additionally meet targets for biodiversity gain. In our case, in adjusting policies so that they meet targets for a 15% gain in biodiversity prevalence, we observe reductions in the flows of monetized ecosystems services delivered by the scheme of up to 20% depending on policy instrument.

Beyond those quantitative findings, our study reveals a number of important qualitative insights. First, we find that pricing by activity tends to lead to schemes that deliver disproportionately on ecosystem service flows that are relatively spatially homogeneous. In our case, that means carbon storage primarily from tree planting. Activity payments are unable to target highly spatially heterogeneous service flows such as recreation and flood mitigation since projects that deliver high values for those service flows are determined as much by their location as by the activity undertaken in that location. Second, we find that optimal policy designs take advantage of patterns of correlation between service flows. In our study, we find that we are prepared to pay a seemingly irrationally high price for one service flow because paying over-the-odds for that service encourages cheaper delivery of some alternative and highly valuable services. The key insight here is that establishing an efficient pricing strategy is complex and may only be achievable through application of the types of optimization technique employed in this research. Finally, we explore how policies might best be designed to accommodate targets for unmonetized ecosystem service flows. Interestingly, in our policy simulations we find that the policy instrument that best delivers on monetized service flows is unable to deliver on our biodiversity targets. The solution to that problem turns out to be quite simple; policies seeking to maximize value from monetized services while reaching targets on unmonetized services should include incentives to deliver on both types of service flow. In our case, when we additionally introduce prices for delivery of improvements in biodiversity prevalence, we are able to identify a pricing strategy that meets the targets while also achieving high levels of value. Biodiversity pricing may, of course, help in ensuring schemes achieve biodiversity targets but such a practice does not obviate the need for development of non-market valuation methodologies that better identify the contribution of biodiversity to society.^9^ Establishing robust values would allow biodiversity to be handled as a monetized service flow in scheme design, ensuring an efficient allocation of investment across different ecosystem services.

We believe our findings to be significant. They reveal that policy design is of first-order importance in determining the efficiency and efficacy of programmes pursuing nature recovery. Well-intentioned, but poorly designed policies for nature recovery may fail to deliver net benefits for society. At the same time, well-designed policies may be highly socially beneficial. That finding alone underscores the critical insights that the natural capital approach, and particularly its extension to the support of policy design, could play in decision-making for nature recovery.

At the same time, our research highlights the fact that the natural capital approach has developed into a sophisticated analytical toolkit that relies on often complex modelling suites embedded in equally complex optimizing frameworks to provide its insights. This results in a significant disconnect. The policy landscape for nature recovery is evolving rapidly. Indeed, across the world, decision-makers are committing to policies that will shape the nature of that recovery over the coming decades. Despite the fact (illustrated by our research) that insights from the natural capital approach could be instrumental in ensuring the success of those policies, those insights are generally out of reach of policy-makers on account of a lack of capacity to develop, interrogate and maintain the sophisticated tools that underpin modern natural capital analysis. While advancing the methods of natural capital research remains important, perhaps the most urgent challenge is to find ways in which the analytical capacity available to the academic community can quickly be made available to those making critical decisions on nature's future.

## Data Availability

The code used in the analyses presented in the paper are available from the public GitHub repository: https://github.com/LEEP-Modelling-Team/Natural-Capital-Modelling-for-Policy-Design-Public [69]. Due to non-disclosure agreements over the input data used in the analysis, that data cannot be shared. Instead a dummy dataset is provided on the GitHub respository that conforms to the structure of the original data allowing use of the code. At the same time, the raw data outputs from each of the analyses as reported in the paper are available on the GitHub repository. Supplementary material is available online [70].

## Endnotes

1 Examples of such ecosystem service modelling tools include ARIES (Artificial Intelligence for Ecosystem Services) [12], Co$tingNature [13], InVEST (Integrated Valuation of Ecosystem Services and Tradeoffs) [14], LUCI (Land Utilisation and Capability Indicator) [15] and MIMES (Multiscale Integrated Model of Ecosystem Services) [16].

2 Note that some of the avenues through which biodiversity delivers value can be monetized. In our study, for example, we place values on the pollination services arising from insect species both in increasing yields of insect-pollinated crops but also in increasing abundance of wildflowers. At the same time, it is worth nothing that some framings of nature conservation object to any sort of monetary valuation of biodiversity's ‘intrinsic value’ [22].

3 To put that sum into context, in 2022 just under £2 billion of public money was channelled to farmers in England. Of that spend, £1.65 billion came in the form of direct payments (unrelated to land use change or delivery of public goods) while a further £290 was allocated through agri-environment schemes [42]. UK government policy is to phase out direct payments by 2027, with increasing emphasis placed on payments to farmers in return for environmental benefits, so-called ‘public money for public goods’ [4].

4 While our paper focuses on the UK policy debate regarding the value-for-money realised by public expenditure, from the perspective of social welfare, payments from the government to farmers that are in excess of costs are simply transfers. In our analyses, those transfers are treated as a cost; in a social welfare analysis they would not be treated as such. Although our methodology readily lends itself to evaluating policies aimed at maximizing social welfare, such an analysis is not the focus of this paper.

5 Our assumption in simulating outcome-based policies is that the payments offered to farmers are calculated in advance of the scheme using scientific modelling tools such as those underpinning NEV. We suspect that the alternative of rewarding farmers only for *ex post* measured changes in outcomes is not feasible. That infeasibility arises both from the complexity of measuring and attributing responsibility for environmental change but also because of the very significant monitoring costs such a scheme would involve. Moreover, presenting farmers with the prospect of signing-up to a contract in which their rewards are uncertain up until the point at which measurements of change are made will likely significantly reduce participation in the scheme.

6 As per footnote 4, in a social welfare analysis one would treat payments to farmers above their costs as a transfer payment rather than a scheme cost. Performing that alternative evaluation of the outcome of this scheme results in an efficiency figure of £0.95 of value for each £1 of real cost. As such, even under a social welfare analysis, the current policy design fails to deliver a scheme that breaks even.

7 More precisely the biodiversity targets were not met in any of 1000 simulations of that policy using a first-come, first-served winner determination rule.

8 We acknowledge that there are many other policy designs that one might adopt beyond pricing by activity and by outcome. One such set of policy instruments are those that forego flat rate prices and instead use competitive tender as a means of allocating funds for habitat creation projects [62,63]. In our research, we examined two such mechanisms and report on their performance in the Supplementary Materials. Alternatively, a number of authors have pointed out the benefits of spatially-differentiating incentive payments in order to direct funds to projects in locations that are more likely to deliver high-value ecosystem services value flows [64]. While we have not explored such policy instruments in this work, their optimal design could be identified using the methods we apply in this work.

9 Examples of recent work in this field include [65–68]
